# Individual Factors Modifying Postoperative Pain Management in Elective Total Hip and Total Knee Replacement Surgery

**DOI:** 10.3390/life14020211

**Published:** 2024-01-31

**Authors:** Alina Jurewicz, Agata Gasiorowska, Katarzyna Leźnicka, Maciej Pawlak, Magdalena Sochacka, Anna Machoy-Mokrzyńska, Andrzej Bohatyrewicz, Agnieszka Maciejewska-Skrendo, Grzegorz Pawlus

**Affiliations:** 1 Department of Specialistic Nursing, Pomeranian Medical University, Żołnierska 48, 71-210 Szczecin, Poland; alina.jurewicz@pum.edu.pl; 2 Faculty of Psychology in Wroclaw, SWPS University of Social Sciences and Humanities, Ostrowskiego 30b, 54-238 Wroclaw, Poland; 3 Department of Physical Education, Academy of Physical Education and Sport, 80-336 Gdansk, Poland; katarzyna.leznicka@awf.gda.pl (K.L.); agnieszka.maciejewska-skrendo@awf.gda.pl (A.M.-S.); grzegorz.pawlus@awf.gda.pl (G.P.); 4 Institute of Physical Culture Sciences, University of Szczecin, 70-453 Szczecin, Poland; magdalena.sochacka@phd.usz.edu.pl; 5 Department of Physiology and Biochemistry, Poznan University of Physical Education, 61-871 Poznan, Poland; pawlak@awf.poznan.pl; 6 Department of Experimental and Clinical Pharmacology, Pomeranian Medical University, 70-111 Szczecin, Poland; anna.machoy-mokrzynska@pum.edu.pl; 7 Department of Orthopaedics Traumatology and Musculoskeletal Oncology, Pomeranian Medical University, Unii Lubelskiej 1, 71-252 Szczecin, Poland; bohatyrewicz@orthopedics.pl

**Keywords:** pressure pain threshold, pressure pain tolerance, total knee replacement, total hip replacement, pain management, morphine, beliefs about controlling pain

## Abstract

Total hip and knee replacements are the most common orthopedic procedures performed due to osteoarthritis. Pain is an intrinsic symptom accompanying osteoarthritis, persisting long before surgery, and continuing during the preoperative and postoperative periods. Appropriate pain management after surgery determines the comfort, duration, and cost of hospitalization, as well as the effectiveness of postoperative rehabilitation. Individual differences in pain perception and tolerance in orthopedic patients remain an important research topic. Therefore, the aim of this study was to investigate the predictors of analgesic requirements (morphine, acetaminophen, and ketoprofen), including individual pain threshold and tolerance, body mass index (BMI), diabetes, and beliefs about pain control in patients undergoing elective hip or knee arthroplasty using a multilevel regression model (N = 147, 85 women, 62 men, 107 after hip replacement, and 40 after knee replacement). Results: Higher pain tolerance was associated with a lower dose of morphine per kg after surgery. Patients undergoing hip surgery received a lower dose of ketoprofen than patients undergoing knee surgery. The more the patient believed in personal pain control, the stronger the negative relationship between pain tolerance and morphine requirement. The lowest doses were given to patients with the highest pain tolerance and the greatest belief in personal control. Factors such as belief in pain control and pain tolerance should be considered in comprehensive postoperative pain management in orthopedic patients to reduce opioid doses and, thus, side effects.

## 1. Introduction

Osteoarthritis (OA) is the most common cause of chronic pain and disability in the musculoskeletal system. Chronic inflammatory or degenerative processes lead to the breakdown of articular cartilage. When the innervated subchondral layer of the bone is exposed, chronic pain occurs, with cytokines and growth factors playing the main role in the pathophysiology of OA [1]. In the UK, approximately 1 in 10 adults suffer from symptomatic, clinically diagnosed OA, with the knee being most commonly affected [2]. In Spain, the prevalence of hip osteoarthritis is 5.13% and that of knee osteoarthritis is 13.83%; these are associated with the female sex, being overweight, and obesity [3]. The incidence of OA increases with age and is estimated to affect 30–50% of adults over the age of 65. Severe degenerative stages of the disease require definitive surgical interventions such as total hip and knee replacements. Data from the United States in 2010 show that these procedures are more common in women than in men [4]. Furthermore, the likelihood of undergoing these operations increases with age. By the age of 80, 5.26% of the population had hip surgery and 10.38% had knee surgery, which equates to approximately 7.2 million people [4]. As the population ages, OA and its treatment are increasingly recognized as significant medical and economic challenges [5,6].

Pain is a highly individual phenomenon as it affects the physical, cognitive, and emotional processes of human life. Defined by IASP in 2020, pain is an unpleasant sensory and emotional experience associated with, or resembling that associated with, actual or potential tissue damage [7,8]. Pain can be seen as a consequence of an imbalance between the classical warning function of the nociceptive system and the descending pain inhibitory pathways, or more generally as an impairment or depletion of the adaptive processes of neuronal plasticity. This set of neurophysiological and neurochemical factors can prevent the development of chronic or neuropathic pain in most cases of nervous system damage. From a physiological point of view, consciously experienced pain is a consequence of the activation of the nociceptive system, but its subjective projection is shaped by a person’s emotional and mental state and is closely related to certain interpersonal relationships and environmental conditions. This is because pain perception is closely related to a person’s emotional and mental state, which is embedded in certain interpersonal relationships and environmental conditions, such as the quality of parenting style, a family environment characterized by hostility and lack of familial warmth, emotional reactivity/neuroticism, low extraversion, catastrophizing, and finally, pain management strategies [9,10,11,12,13,14,15]. Pain management has a significant impact on clinical outcomes, including pain intensity and disability [14]. Pain management after surgery usually follows standards and rules that should lead to the elimination or alleviation of pain [16,17]. This effect is not always achieved, and the individual response to analgesic treatment remains unsatisfactory [18]. This may be due to the complex mechanisms of pain in OA, its cause and nature, as well as other individual influencing factors such as concomitant diseases, especially diabetes, obesity, cancer, or mental illness [19,20]. Certainly, a comprehensive understanding of these individual factors will help to increase the effectiveness of pain management and individualize the approach within the standards.

Numerous researchers describe the risks and predictive factors of postoperative pain after musculoskeletal surgery [21,22], but this assessment appears to be far from complete. Therefore, the aim of this study was to investigate the predictors of analgesic requirements in patients undergoing elective hip or knee arthroplasty. Specifically, in this paper, we analyze (1) individual pain sensitivity before surgery and (2) beliefs about pain control as predictors of pain sensitivity after surgery, operationalized as required morphine doses in orthopedic patients and pain management after surgery. We anticipate that these results will help to establish appropriate criteria for the assessment of analgesic benefits and improve the efficacy of analgesic dosing.

## 2. Material and Methods

### 2.1. Study Recruitment

The study group was recruited in the Department of Orthopedics, Traumatology, and Musculoskeletal Oncology of Pomeranian Medical University. We included all patients who were admitted to the clinic between January and September 2020, consented to participate in the study, and were eligible for surgery. Based on their clinical condition and imaging studies, patients met the criteria for arthroplasty and were eligible for total hip arthroplasty (THR) or total knee arthroplasty (TKR). We included patients with moderate or severe radiographic changes according to the classification of Kellgren and Lawrence [23], i.e., grades 3 and 4. The implant used in THR was an uncemented Pinnacle^®^ cup and Corail^®^ stem (DePuy Synthes, Warsaw, IN, USA). All procedures were performed using the anterolateral approach as described by Watson-Jones [24], by two experienced hip surgeons. The implant used in the TKR was a cemented Vanguard^®^ knee (Biomet, Inc., Warsaw, IN, USA). All procedures were performed using the medial parapatellar approach [25] by two experienced knee arthroplasty surgeons. Midazolam (0.05 mg/kg) was used for anesthetic premedication in all cases. Patients undergoing THR and TKR received subarachnoid anesthesia with bupivacaine 0.5% (0.15 mg/kg) after the L2/L3 levels were determined for hip and knee arthroplasty [26]. The average length of stay in hospital was 3.6 days (range 3–5 days).

### 2.2. Procedure

The participants were informed about the purpose of the study and gave their written consent to participate in the research. The study was approved by the bioethics committee of the Regional Medical Board (KB–10/19).

At the time of admission to the hospital, all patients declared that they were fasting and had not taken any painkillers. On the first day of hospitalization, participants were asked to complete a personal demographic questionnaire and a Polish adaptation of the Beliefs about Pain Control Questionnaire [27,28]. After these initial tasks, on the same day (before surgery), we measured pressure pain threshold and tolerance using the methods described later in this article.

All patients undergoing total hip and knee arthroplasty received routine anesthetic premedication and postoperative analgesia. After surgery, the method of treatment was analyzed, taking into account the type of analgesic administered, single and daily doses, route of administration, and refusal to administer the drug due to lack of pain. The efficacy of the analgesic treatment was assessed using the numerical rating scale (NRS), a one-dimensional assessment of pain intensity (PI). The scale was presented to patients, and they were asked to rate their pain on a scale of 0 to 10, with 0 representing no pain and 10 representing the worst pain imaginable [29]. The original cut-off points for the NRS were mild (1–4), moderate (5–6), and severe pain (7–10) [30]. The mild pain group was divided into two subgroups, 1–3 points and 4 points [31]. Pain intensity was registered in the clinical sheet by qualified nurses being on duty, 6 times a day (every four hours—4 a.m., 8 a.m., 12 a.m., 4 p.m., 8 p.m., 12 p.m.), beginning after arthroplasty and continuing for 48 h. The medicaments were administered in a single-blind procedure based on a typical pain management plan, as described below. Patients who rated their pain as 1–3 points were prescribed 1 g of acetaminophen intravenously 1–4 times a day when needed [32]. For mild pain rated at 4 points, an additional 0.1 g of the non-steroid ketoprofen was administered intravenously 1–2 times daily as required [33]. For moderate pain (PI > 4), subjects received 0.01 g of intravenous morphine up to four times daily until the PI was reduced to 4 or less [34]. Our predefined outcome measures to assess postoperative pain were acetaminophen, non-steroid ketoprofen, and morphine consumption taken on the first postoperative day. All patients in our study required medications and, hence, received one or more analgesics, with *n* = 106 (72.1% of the sample) receiving all three substances.

### 2.3. Measurements

#### 2.3.1. Pain Threshold and Tolerance

Pressure pain threshold (PPT—the lowest intensity of a given stimulus: sound, heat, touch, at which a person begins to feel pain) and pressure pain tolerance (PTOL—the greatest pain stimulus a person can tolerate) were measured using a standard FPN 100 Algometer (Wagner Instruments, Greenwich, CT, USA) with a measurement range of 0 to 20 kg and an attached disk-shaped rubber tip of 1 cm^2^. Pain threshold and tolerance measurements were made on an interval scale with one decimal place. Although the measurement capacity of the device was limited to 20 kg, none of the participants exceeded this threshold and could tolerate a pressure of more than 20 kg/cm^2^.

Each participant was informed of the diagnostic significance of these measurements and instructed on how to behave during the test. Each measurement was preceded by two trials to ensure that the patient could distinguish the sensation of pressure from pain and was able to stop the pressure measurement at the correct moment. The measurements were taken at 10-second intervals on both upper limbs on the dorsum of the hand between the thumb and index finger. The experimenter placed the algometer head on the area to be measured and applied uniform stimuli at a rate of 50 kPa/s (0.5 kg/s). The pain felt by the individual was expressed by the command “stop”, followed by a recording of the pressure level, referred to as the pressure pain threshold (PPT). The test continued until the subject could no longer tolerate the intensity of the stimulus and signaled the end of the measurement, which was then classified as pain tolerance (PTOL).

#### 2.3.2. Beliefs about Pain Control Strategies

To assess patients’ attitudes toward pain control, we used the Beliefs about Pain Control Questionnaire (BPCQ) [27] in its Polish adaptation by Juczyński [28]. This scale was developed to measure pain-related beliefs in patients with pain and those who do not currently have this symptom. The BPCQ consists of 13 statements with a 6-point scale ranging from 1 = “strongly disagree” to 6 “strongly agree”. These items measure the three dimensions of beliefs about the control of pain: (1) internal or personal control, (2) influence of others (powerful others, namely physicians, control pain), (3) pain is controlled by random events [27,28]. Patients’ responses were averaged within each dimension. The higher the score in a particular domain, the stronger the belief that pain can be controlled by a particular factor [27,35].

### 2.4. Statistical Analysis

Data analysis was performed using JASP (descriptive statistics) and JAMOVI (multilevel linear regression) [36,37,38]. The threshold for statistical significance was set at *p* < 0.05. The data were considered nested because all participants took two measurements of pain threshold and pain tolerance and because some of the participants underwent knee replacement while others underwent hip replacement. Therefore, we used multilevel regression with JAMOVI using REML estimation, which allows the use of variables that deviate from the normal distribution, with data nested within participants and types of surgery, with random intercepts for the type of surgery. In the multilevel regression in step 1, we regressed a dose of morphine per kg as the DV on PPT and PTOL as our main predictors. However, age, diabetes diagnosis, BMI, and doses of other analgesics administered after surgery could affect the relationship between pain sensitivity and the required morphine dose. Therefore, in step 2, we included these covariates in the multilevel regression to show that the negative relationship between pain tolerance and morphine dose persisted after controlling for confounding factors. Finally, the main aim of this work was to investigate the interplay between objective pain sensitivity (pain tolerance) and psychological factors (beliefs about pain control strategies). Therefore, in step 3, we introduced three dimensions of beliefs about pain control as additional predictors and also considered their interactions with pain threshold and pain tolerance.

## 3. Results

### 3.1. Study Population and Sensitivity Analysis

The prospective study was performed on 147 patients hospitalized for hip or knee replacement surgery (85 women and 62 men; age M = 62.39, SD = 9.67, mdn = 65, range 33–76). The mean BMI of the participants was M = 30.02 (SD = 4.69, mdn = 29.80, range 20.50–43.40), and 32 of them (21.77%) had been diagnosed with diabetes type 2 with regularly controlled glucose levels. Sensitivity analysis with G*Power [39] showed that with a power of 1 − β = 80% and a significance of α = 0.05, such a sample is large enough to detect an effect of R^2^ = 0.05 in a regression analysis. Therefore, our sample size was large enough to detect even weak associations between our variables of interest.

### 3.2. Descriptive Analyses and Correlations

Table 1 shows the descriptive statistics for the whole sample and compares the two types of operations. We found no differences between participants undergoing hip and knee surgery in terms of pain threshold, pain tolerance, beliefs regarding pain control strategies, and doses of acetaminophen and ketoprofen administered. The only significant difference was in the dose of ketoprofen: patients undergoing hip surgery received a lower dose than those undergoing knee surgery. The proportion of men and women did not differ by type of surgery, χ^2^(1) = 2.11, *p* = 0.146; similarly, the proportion of patients with diagnosed diabetes, χ^2^(1) = 0.337, *p* = 0.562, and their mean age did not differ significantly. The average BMI was higher in patients with knee replacements than in those with hip replacements (see Table 1).

Table 2 shows the correlations between the variables of interest. Participants’ age correlated with beliefs about pain control (powerful others, personal control, and random happenings). We found that older participants scored higher on all dimensions than younger participants, with the strongest correlation for the belief that others can control pain. This group of participants was also more likely to be diagnosed with diabetes than younger participants. Pain thresholds and pain tolerance on both hands were highly correlated in all patients. Pain thresholds in the dominant and non-dominant hand and pain tolerance in the non-dominant hand were positively correlated with the belief that they could control the pain themselves. Receiving higher doses of ketoprofen was associated with receiving higher doses of acetaminophen and morphine, but doses of the latter two analgesics were not correlated. The three dimensions of beliefs about pain control strategies were also positively correlated but with low to moderate strength.

### 3.3. Multilevel Regression Analysis

As multiple correlations occurred between the predictor variables examined, we conducted a multilevel regression analysis rather than relying on the raw correlations. First, a multilevel analysis was performed with pain tolerance and pain threshold as predictors of morphine dose per kg (step 1 in Table 3). Pain tolerance before surgery was a significant predictor of the amount of morphine administered per kg after surgery, such that higher pain tolerance was associated with lower morphine doses. In step 2 of the analysis (Table 3), potential confounders were added, including a diabetes diagnosis, age, BMI, and the amounts of other analgesics administered after surgery. A significant and negative association was found between patients’ BMI and morphine dose, such that participants with a higher BMI received a lower dose per kg. In addition, we found a significant and positive effect of acetaminophen, i.e., the more acetaminophen and the fewer ketoprofen patients received after surgery, the higher the morphine dose administered afterward. Most importantly, however, the relationship between pain tolerance and morphine dose remained significant even after controlling for these additional variables.

Finally, three variables representing the perceived sources of pain control were added in step 3 (Table 3): namely, personal control over pain, control by powerful others, and control by random events, as well as the interactions between these three variables and two variables representing pain sensitivity (pain threshold and pain tolerance). After adding these variables, the main effects of BMI and acetaminophen intake remained significant, as did the main effect of pain tolerance. A significant interaction between PTOL and personal control was also observed. No other interactions were significant.

**Figure 1:**
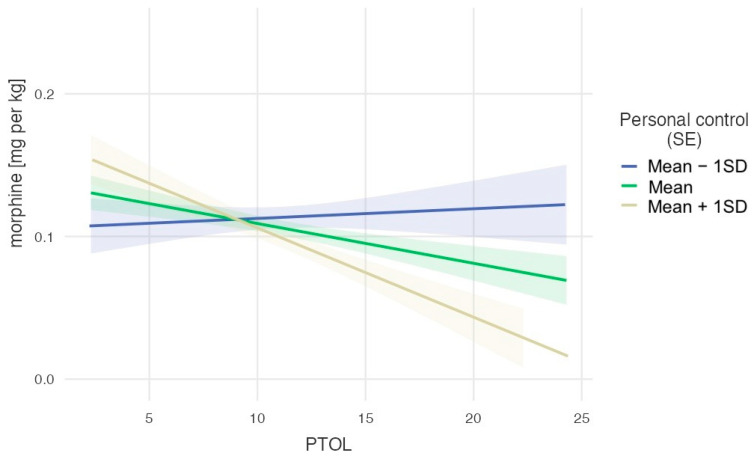
The dose of morphine per kg as a function of pain tolerance for three levels of belief in personal factors controlling pain.

To decompose the observed significant interaction, we used a pick-a-point approach and examined the effect of one variable on three levels of the other variable (M − 1SD, M, M + 1SD). First, we tested the relationship between pain tolerance and morphine dose per kg after surgery as a function of the belief in personal control over pain perception (Figure 1). The effect of PTOL on morphine dose per kg was not significant for the lowest levels of the moderator, M − 1SD = 2.45, b = −0.001, se = 0.002, t(277.52) = 0.33, *p* = 0.740. This effect was negative and weak but significant for the medium level of the moderator, M = 3.41, b = −0.003, se = 0.001, t(277.62) = −2.26, *p* = 0.024, and the strongest and negative for the highest level of the moderator, M + 1SD = 4.37, b = −0.006, se = 0.002, t(276.45) = −3.47, *p* < 0.001. These results suggest that people with a low belief in personal control over pain received the highest doses of morphine regardless of their pain tolerance measured prior to surgery. The higher the belief in personal control over pain, the stronger the relationship between pain tolerance and morphine requirement, so the lowest doses were given to those with the highest pain tolerance and highest belief in personal control.

**Figure 2:**
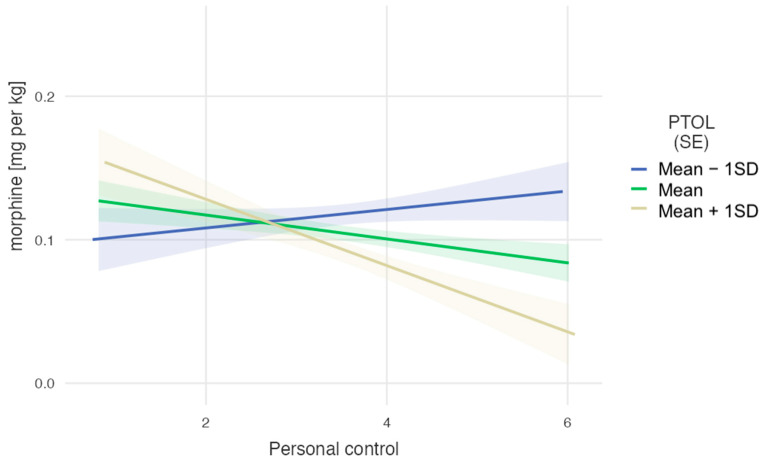
The dose of morphine per kg as a function of belief in personal factors controlling pain for three levels of pain tolerance.

An alternative decomposition of the same interaction (Figure 2) revealed that participants with the lowest PTOL (M − 1SD = 7.20) required the highest doses of morphine, regardless of their perceived personal control, b = 0.006, se = 0.008, t(276,79) = 0.82, *p* = 0.411. For those with an average PTOL level (M = 11.30), the effect of personal pain control was also not significant, b = −0.008, se = 0.005, t(276.79) = −1.72, *p* = 0.087. However, for those with a high PTOL level (M = 15.39), the effect of perceived personal control was significant and negative, b = −0.023, se = 0.008, t(276.79) = −2.86, *p* = 0.005. These results suggest that people with a low pain tolerance received the highest doses of morphine regardless of their belief in personal control over pain measured prior to surgery. The higher the pain tolerance, the stronger the relationship between belief in personal control over pain and morphine requirements, so the lowest doses were given to those with the highest pain tolerance and highest belief in personal control.

## 4. Discussion

Pain is an intrinsic concomitant symptom of osteoarthritis and one of the factors influencing one’s decision to undergo surgery; it also occurs in the postoperative phase. Adequate pain management has an impact on the comfort, duration, and cost of hospitalization, as well as on the effectiveness of postoperative rehabilitation. When observing patients treated with analgesics (opioids as well as acetaminophen and non-steroidal anti-inflammatory drugs), inter-individual differences in effectiveness can be observed. Despite the conversion of the drug dose per kilogram of body weight, identical administration regimens, and the exclusion of the risk of drug–drug interactions, patients respond differently to treatment. The degree of pain intensity, the risk of conversion from acute to chronic pain, and the effectiveness of therapy—both for acute and post-operative or post-traumatic pain—may depend on many factors.

Some studies suggest that older people have lower pain sensitivity and better pain tolerance than their younger counterparts, but the research findings in this area are inconclusive [40,41]. In our research, we found no correlations between age and the two indicators of pain perception (pain threshold and pain tolerance), but this may be due to the relative homogeneity of our sample regarding age (only 11.6% of the sample was under 50 years of age). However, very interesting observations relate to the correlation between patients’ age and beliefs about pain control. Older participants had higher scores on all dimensions than younger participants, with the strongest correlation for the belief that other people can control pain. In addition, our further analysis (Table 3) revealed that beliefs about pain control were not directly related to morphine doses received after surgery, but that beliefs about personal pain control moderated the effects of pain tolerance, such that individuals with low beliefs about personal pain control received higher doses of morphine regardless of their pain tolerance.

When analyzing the results of body mass index (BMI), we found no significant correlation of this factor with pain perception, but we also found a weak negative association with the dose of morphine per kg after controlling for other confounds. These inconclusive results may be due to the relative homogeneity of our sample regarding BMI (only 16% of our participants had a BMI under 25). Interestingly, Emerson, et al. [42] found no differences between individuals with different BMI in pain sensitivity to noxious heat and cold stimuli and no associations between central adiposity or body fat and pain responses to noxious heat or cold stimuli. In contrast, research by Torensma et al. [43] showed that individuals with severe obesity exhibited hypoalgesia to noxious electrical stimuli and had difficulty distinguishing between pain threshold and tolerance in response to experimental noxious thermal and electrical stimuli. In overweight or obese patients with knee osteoarthritis, intervention with diet and exercise resulted in a statistically significant but small difference in knee pain over an 18-month period (compared with the control condition); however, the magnitude of the difference in pain between the groups is of uncertain clinical significance [44].

Although diabetic polyneuropathy is the most common microvascular complication, in our studies, we found no significant correlations between diabetes and pain threshold and pain tolerance and no association between diabetes and the dose of morphine received on the first-day post-surgery. Akintoye et al. [45] showed that patients with diabetic neuropathy have a poor endogenous opioid peptide system associated with increased pain perception and high insulin resistance. Interestingly, the studies by Telli et al. [46] showed that diabetic patients with neuropathy had the highest pain threshold and tolerance and that the impairment of light touch sense was higher in diabetic patients with neuropathy than in healthy participants. In another experimental study, glucose-induced hyperglycemic rats were found to have a significantly higher pain threshold than control animals [47].

Sokil et al. [48] demonstrated that a higher qualitative pain threshold was associated with lower postoperative opioid consumption, but this did not reach statistical significance as an independent predictor for the top quartile of pill users. In our study, pre-treatment pain tolerance scores were significant predictors of the amount of morphine administered per kg after surgery, such that higher pain tolerance was associated with lower morphine doses.

Opioids, which are used in the pharmacological treatment of postoperative pain, are undoubtedly an effective means of relieving acute and chronic pain of various causes. However, in addition to their analgesic effects, they also influence behavior and mood, respiratory system function, the cardiovascular system, the gastrointestinal tract, and the neuroendocrine and immune systems [49]. The immunosuppressive effect of opioids may increase the risk of infection in the postoperative period, as well as the possibility of opioid-induced hyperalgesia, i.e., the so-called opioid paradox [50]. It is still debated but generally accepted that opioids, although they can cause significant side effects, remain important components of postoperative pain management [51]. Due to the wide range of side effects of opioid analgesics (morphine was used in the present study), methods to reduce the opioid dose while ensuring adequate efficacy of postoperative pain management and improving patient comfort should be encouraged. In addition, the risk of complications is lower, and the length of hospital stay is shorter [49,52].

One of the most commonly used non-opioid analgesics is acetaminophen. Its use is associated with a reduction in pain intensity and, thus, it offers the possibility of reducing the dose of opioid analgesics and reducing the severity and frequency of opioid side effects. It should be emphasized that acetaminophen is preferably administered intravenously in the first days after surgery, which, due to its PK/PD (pharmacokinetics/pharmacodynamics) profile, allows a higher concentration of the drug, which translates into better efficacy of analgesic treatment, especially since the pharmacokinetics of acetaminophen is linear [53]. Since acetaminophen acts at all levels of pain stimuli, starting from the receptors in the tissues and through the spinal cord to the thalamus and cerebral cortex where pain sensations are received, the mechanism of its analgesic action is complex. Several possibilities are still under consideration, including influencing both peripheral antinociceptive processes (by inhibiting COX activity) and central antinociceptive processes (through COX inhibition, impact on serotonergic descending inhibitory pathways, L-arginine/NO pathway, cannabinoid system), as well as “oxidoreductive” mechanisms [53].

Non-steroidal anti-inflammatory drugs (NSAIDs) are also used to relieve postoperative pain, including ketoprofen, which was used in the present study. In addition to their analgesic and antipyretic effects, drugs from this group also have anti-inflammatory effects. In addition to inhibiting prostaglandin synthesis, they can also influence other important pathophysiological processes involved in the development of inflammatory nociceptive pain, for example, by inhibiting the expression of inducible nitric oxide synthase, inhibiting the activation of NF-kappa B, activating the lipoxin system, and inhibiting substance P activity. In addition, the effects of NSAIDs may be due to the activation of supraspinal cholinergic pathways and the activation of the endogenous opioid peptide system. Ketoprofen is a drug that is 99% bound to plasma proteins and is intensively metabolized (conjugated to glucuronic acid). Its use, similar to acetaminophen, allows a reduction in opioid doses and thus the possibility of using it in multimodal analgesia. NSAIDs have the potential to play an important role in reducing postoperative opioid requirements [54,55]. Reducing opioid use is expected to reduce opioid-related side effects and help reverse the opioid epidemic [49]. The preventive use of a multimodal pain management approach has become the standard of care for pain management after hip and knee arthroplasty [55].

As was also shown in this study, patients’ beliefs about their own ability to control pain have a significant impact on their overall pain experience [56]. In our study, patients with a lower belief in their ability to control pain received higher doses of morphine, regardless of their pain tolerance prior to surgery. Conversely, patients with a stronger belief in their personal control over pain showed a direct correlation between their pain tolerance and the morphine dose received—the greater the pain tolerance and belief in personal control, the lower the morphine dose administered. This underlines the importance of psychological factors in pain management and suggests that strengthening patients’ beliefs in their own control over pain could potentially lead to a reduction in the need for opioid analgesics. Psychological treatment aimed at changing patients’ beliefs about the causes and threat value of pain can lead to significant and lasting pain relief in people with chronic low back pain [57]. Psychological modeling of beliefs about pain control may be a valuable way to improve overall clinical outcomes [58]. Interestingly, acceptance and commitment therapy could be used as psychological interventions alongside pharmacotherapy to improve pain acceptance and reduce pain perception in patients with painful diabetic neuropathy [59], which could reduce the need for analgesics, including opioids. Exercise programs, patient education, and cognitive behavioral therapies for patients with chronic pain can reduce maladaptive behaviors and improve positive lifestyle choices, which could have a direct impact on clinical outcomes [18].

In addition, the right approach to pain management in the postoperative period can allow patients to engage in appropriate physical activity, improving the quality of functioning [60,61]. There is also a factor that should influence pain management, i.e., moderate physical activity, which has a curative effect by increasing the pain threshold and tolerance [62,63]. As a modifier of analgesic therapy, it should be the subject of further research.

The study presented here has some limitations that should be noted. First, the study population included patients with hip and knee osteoarthritis who underwent different surgical procedures. These differences in the type of surgery could potentially influence the perception of pain and, thus, the use of pain medication. In addition, there is evidence in the literature that pain is perceived, described, and evaluated differently, with factors such as cultural differences possibly playing a role. These differences could lead to different patterns of medication use. Although the sample size of 147 patients was sufficient to analyze the effective doses of pain medication, it limited the scope for a comprehensive statistical analysis. We believe that larger studies would be more effective to thoroughly investigate all the nuances of these associations.

## 5. Conclusions

Our findings suggest that assessing preoperative pain tolerance and identifying a patient’s beliefs about pain control may be effective predictive factors in determining individual analgesic doses. These factors and their interrelationships have a significant impact on postoperative pain experience and effective doses during treatment. Patients with less confidence in pain control required more morphine, regardless of pain tolerance before surgery. Those with higher confidence in pain control and higher pain tolerance required less morphine. This emphasizes the psychological component of pain management and suggests that increasing confidence in self-control can reduce opioid dependence. The effects of patients’ age, body weight, and concomitant diseases require further research. Postoperative pain management in hip and knee replacement surgery is a multidimensional challenge influenced by several patient-specific factors. A personalized, holistic approach that takes these factors into account is essential to optimize pain relief, reduce complications, and promote successful postoperative recovery.

## Figures and Tables

**Table 1:** Descriptives for the variables measured in the study, along with comparisons across types of surgery.

Variable	Min	Max	M	Mdn	SD	Hip Replacement *n* = 107		Knee Replacement *n* = 40		Comparison between Types of Surgery	
						M	SD	M	SD	t(145)	Cohen’s *d*
Age	33	76	62.39	65.00	9.67	62.07	10.43	63.27	7.32	−0.67	−0.12
BMI	20.5	43.4	30.02	29.8	4.69	29.00	4.37	32.76	4.46	−4.62 ***	−0.86
PPT dominant	2.80	23.20	7.17	7.10	2.60	6.99	2.51	7.67	2.79	−1.42	−0.26
PPT non-dominant	3.30	24.00	6.63	6.30	2.44	6.51	2.29	6.93	2.81	−0.92	−0.17
PTOL dominant	1.90	14.90	11.40	10.90	4.09	11.28	3.99	11.72	4.38	−0.59	−0.11
PTOL non-dominant	1.80	15.20	11.20	10.40	4.11	11.23	4.02	11.10	4.40	0.17	0.03
Acetaminophen (in 1000 mg)	0	4.00	2.31	2.00	0.83	2.29	0.86	2.35	0.77	−0.39	−0.07
Ketoprofen (in 100 mg)	0	2.00	1.35	1.00	0.56	1.29	0.57	1.52	0.51	−2.30 *	−0.43
Morphine (in mg)	0	20.00	8.20	10.00	5.70	7.99	5.66	8.75	5.86	−0.72	−0.13
Morphine (in mg/kg)	0	0.30	0.10	0.11	0.07	0.10	0.07	0.10	0.07	0.12	0.02
Powerful others	1	6	4.25	4.25	1.12	4.21	1.09	4.37	1.19	−0.79	−0.15
Personal control	1	6	3.48	3.50	0.96	3.36	0.95	3.56	1.01	−1.14	−0.21
Random events	1	6	3.41	3.60	0.96	3.44	1.00	3.58	0.87	−0.78	−0.14

PPT—pain threshold, PTOL—pain tolerance. Note: *** *p* < 0.001, * *p* < 0.05

**Table 2:** Correlations between variables measured in the study.

Variable	1		2		3		4		5		6		7		8		9		10		11		12	
1. Age	—																							
2. BMI	0.05		—																					
3. Diabetes (1 = yes, 0 = no)	0.20	**	0.16	*	—																			
4. PPT dominant hand	0.01		0.09		0.01		—																	
5. PPT non-dominant hand	−0.02		0.02		−0.04		0.70	***	—															
6. PTOL dominant hand	−0.05		−0.08		−0.08		0.63	***	0.60	***	—													
7. PTOL non-dominant hand	−0.07		0.03		−0.05		0.57	***	0.64	***	0.80	***	—											
8. Ketoprofen (in 100 mg)	0.07		0.06		0.01		−0.07		0.01		−0.08		0.02		—									
9. Acetaminophen (in g)	0.15		−0.05		0.06		−0.01		0.03		0.04		0.01		0.28	***	—							
10. Morphine (in mg/kg)	0.05		−0.14		0.03		−0.10		0.01		−0.13		−0.14		0.26	**	−0.01		—					
11. Powerful others	0.17	*	0.15		0.20	**	0.18	*	0.20	*	0.10		0.17	*	0.15		0.09		−0.03		—			
12. Personal control	0.35	***	0.20	*	0.14		0.07		0.08		0.02		0.05		0.15		0.09		0.09		0.27	***	—	
13. Random events	0.21	*	0.13		0.11		0.05		0.04		0.06		0.05		0.14		0.11		0.10		0.44	***	0.44	***

PPT—pain threshold, PTOL—pain tolerance. Note: *** *p* < 0.001, ** *p* < 0.01, * *p* < 0.05; Person correlations for continuous variables, tau-b Kendall correlation for diabetes.

**Table 3:** Predictors of the dose of morphine (mg per kg). Results of multilevel regression.

	Step 1					Step 2					Step 3				
Predictors	b	SE	df	t		b	SE	df	t		b	SE	df	t	
(Intercept)	0.13	0.01	291.00	8.76	***	0.13	0.01	35.42	8.94	***	0.12	0.06	262.88	0.35	
PTOL	0.003	0.001	291.00	−2.26	*	0.00	0.00	285.60	−2.31	*	0.01	0.01	276.60	−2.26	
PPT	0.001	0.002	291.00	0.72		0.00	0.00	285.67	1.03		0.00	0.01	277.00	1.27	
Diabetes						0.01	0.01	285.70	1.47		0.02	0.01	276.87	1.61	
Age						<0.001	<0.001	285.10	0.45		<0.001	<0.001	276.07	0.00	
BMI						−0.003	0.00	71.22	−3.48	***	-0.002	0.001	52.46	−2.56	*
Ketoprofen (in 100 mg)						−0.01	0.01	216.35	−1.91		−0.01	0.01	185.26	−1.21	
Acetaminophen (in g)						0.03	0.01	285.85	5.24	***	0.02	0.01	277.00	4.25	***
Powerful others											0.002	0.004	276.81	0.37	
Personal control											−0.01	0.01	276.63	−1.72	
Chance happenings											0.01	0.01	276.01	1.43	
PTOL × Powerful others											-0.003	0.002	276.36	1.84	
PTOL × Personal control											<0.001	0.002	276.55	−2.37	*
PTOL × Random events											<0.01	0.001	276.74	−0.07	
PPT × Powerful others											-0.003	0.002	276.71	−1.53	
PPT × Personal control											<0.001	0.002	276.97	0.08	
PPT × Random events											0.001	0.003	276.07	0.38	
R^2^	2.00%					13.18%					17.78%				

PPT—pain threshold, PTOL—pain tolerance Note: *** *p* < 0.001, * *p* < 0.05.

## Data Availability

The data are available upon request from the authors.
